# Isolated Lip Angioedema Associated With Spironolactone: A Case Report

**DOI:** 10.7759/cureus.115347

**Published:** 2026-08-28

**Authors:** Tawfeeq Hosein, Christopher Chang, Sriram Nallamothu, Nalini D Maharaj

**Affiliations:** 1 Department of Primary Health Care, South-West Regional Health Authority, San Fernando, TTO; 2 Department of Internal Medicine, South-West Regional Health Authority, Point Fortin, TTO; 3 Department of Primary Health Care, South-West Regional Health Authority, Penal, TTO

**Keywords:** adverse drug reaction, angioedema, drug-induced angioedema, hypersensitivity reaction, lip edema, positive dechallenge, spironolactone

## Abstract

Spironolactone is a widely used mineralocorticoid receptor antagonist with a well-established adverse-effect profile; however, angioedema is uncommon and a poorly recognized adverse reaction.

We report a case of suspected isolated lip angioedema associated with spironolactone therapy in a 74-year-old woman. The patient presented with a two-week history of persistent upper-lip swelling and facial heaviness without urticaria, pruritus, tongue or oropharyngeal involvement, or symptoms of airway compromise. Although spironolactone had been prescribed approximately one year earlier, the patient had been poorly adherent and had resumed consistent daily use approximately one month before the onset of symptoms. She continued spironolactone despite the development of swelling. Clinical evaluation and laboratory investigations did not identify an alternative cause, and serum complement C4 was within the normal range. C1-esterase inhibitor antigenic and functional assays were unavailable. Spironolactone was discontinued, resulting in substantial improvement by Day 7 and complete resolution by Day 14 without recurrence. The Naranjo Adverse Drug Reaction Probability Scale yielded a score of 6, consistent with a probable adverse drug reaction.

This case highlights isolated lip angioedema as a potential, although rare, adverse reaction to spironolactone and underscores the importance of establishing actual medication exposure when assessing suspected drug-related reactions. Recognition of uncommon medication triggers may facilitate timely withdrawal of the offending agent and prevent potentially more severe recurrence.

## Introduction

Angioedema is a localized, transient swelling of the deeper layers of the skin and mucosal tissues resulting from increased microvascular permeability [1,2]. It most commonly affects the lips, tongue, face, and upper airway and may occur as an isolated phenomenon or in association with urticaria and other manifestations of hypersensitivity [1]. Although many episodes are self-limiting, involvement of the tongue, pharynx, or larynx can result in life-threatening airway obstruction, making prompt recognition and assessment essential [2].

Drug-induced angioedema is an important cause of acute facial and oropharyngeal swelling. Angiotensin-converting enzyme inhibitors (ACEi) are among the best-recognized pharmacological triggers, with angioedema also reported with other medications, including angiotensin receptor blockers (ARBs), non-steroidal anti-inflammatory drugs (NSAIDs), and aspirin [1].

Spironolactone is a widely used mineralocorticoid receptor antagonist indicated for conditions including hypertension, heart failure, oedema, and hyperaldosteronism [3]. Its adverse-effect profile is well established and includes hyperkalemia, renal dysfunction, and endocrine-related effects [3]. Although angioedema has been reported in association with spironolactone, detailed clinical reports of isolated lip angioedema remain scarce. Pharmacovigilance data provide limited evidence of a potential association; one analysis identified spironolactone in 19 cases (2.1%) of reported angioedema associated with renin-angiotensin system-related medications [4]. However, spontaneous adverse-event reports are subject to reporting bias, incomplete clinical information, and confounding, and cannot establish a causal relationship between drug exposure and angioedema [4]. More recently, an analysis of the US Food and Drug Administration Adverse Event Reporting System identified 8,566 reports in which spironolactone was the primary suspect drug across 2,409 adverse-event preferred terms; notably, angioedema did not emerge among the leading reported adverse-event signals [5]. Spironolactone has nevertheless been associated with hypersensitivity reactions, including drug reaction with eosinophilia and systemic symptoms (DRESS) and other immune-mediated cutaneous reactions [6-8], providing evidence that immune-mediated adverse reactions to the drug are biologically plausible. Collectively, the available evidence suggests that spironolactone-associated angioedema may occur but appears to be uncommon, while the paucity of detailed clinical reports limits characterization of this potential adverse reaction.

The diagnosis of drug-associated angioedema is primarily clinical and requires careful consideration of alternative causes, including allergic reactions, other medication-related reactions, hereditary or acquired angioedema, infection, and local inflammatory conditions. Establishing a causal relationship with an individual medication may be particularly challenging when the suspected drug is not a commonly recognized trigger. A clear temporal relationship, improvement following withdrawal of the suspected medication, exclusion of competing diagnoses, and absence of recurrence following discontinuation can provide important supporting evidence for possible drug causality.

We present a case of isolated lip swelling associated with spironolactone therapy, with clinical improvement following discontinuation of the medication. This case highlights the importance of considering less commonly recognized medications as potential causes of angioedema. It emphasizes the role of a detailed medication history and systematic evaluation of alternative causes in patients presenting with persistent lip swelling.

## Case presentation

A 74-year-old woman presented to her primary care physician with a two-week history of persistent and isolated lip swelling associated with a sensation of facial heaviness. The patient reported mild tingling and a sensation of warmth over the affected area; however, denied any swelling of the tongue or throat and had no associated pain, pruritus, burning sensation, or facial numbness. She denied the presence of any symptoms associated with airway compromise, as she did not experience any dyspnea, dysphagia, dysphonia, hoarseness, throat tightness, drooling, wheezing or stridor. Further consultation revealed no associated rash, urticaria, facial flushing, fever, oral ulceration, dental pain or purulent oral discharge.

Her medical history was significant for a 10-year history of dyslipidemia, which was well controlled with rosuvastatin 20 mg orally at night. She was diagnosed with atrial cardiomyopathy in May 2025, for which her cardiologist prescribed spironolactone 25 mg orally once daily. The patient reported that she had been non-adherent to spironolactone for approximately one year but resumed consistent daily use in June 2026. Approximately one month after resuming regular therapy, she developed persistent lip swelling. Despite the onset of symptoms, she continued taking spironolactone at the prescribed dosage until presentation to the health facility in July 2026.

At the time of presentation, her only prescribed medications were rosuvastatin 20 mg orally once nightly and spironolactone 25 mg orally once daily. She reported no use of ACEi, ARBs, NSAIDs, over-the-counter preparations, or herbal supplements. She had no known drug or food allergies and no previous history of angioedema or recurrent facial swelling. She had never required emergency department assessment, hospital admission, or adrenaline administration for allergic reactions. There was no family history of angioedema, recurrent unexplained swelling, hereditary C1 esterase inhibitor deficiency or sudden death secondary to airway compromise. She denied cigarette smoking, alcohol consumption, or recreational drug use. There was no history of recent travel, infections, dental procedures, insect bites, trauma, vaccination or ingestion of unfamiliar foods before symptom onset.

On examination, the patient appeared comfortable and in no apparent distress. She was able to speak in full sentences without respiratory difficulty. Vital signs were stable: temperature 36.1°C, blood pressure 129/82 mmHg, heart rate 80 beats per minute, respiratory rate 18 breaths per minute, and oxygen saturation 99% on room air.

**Figure 1:**
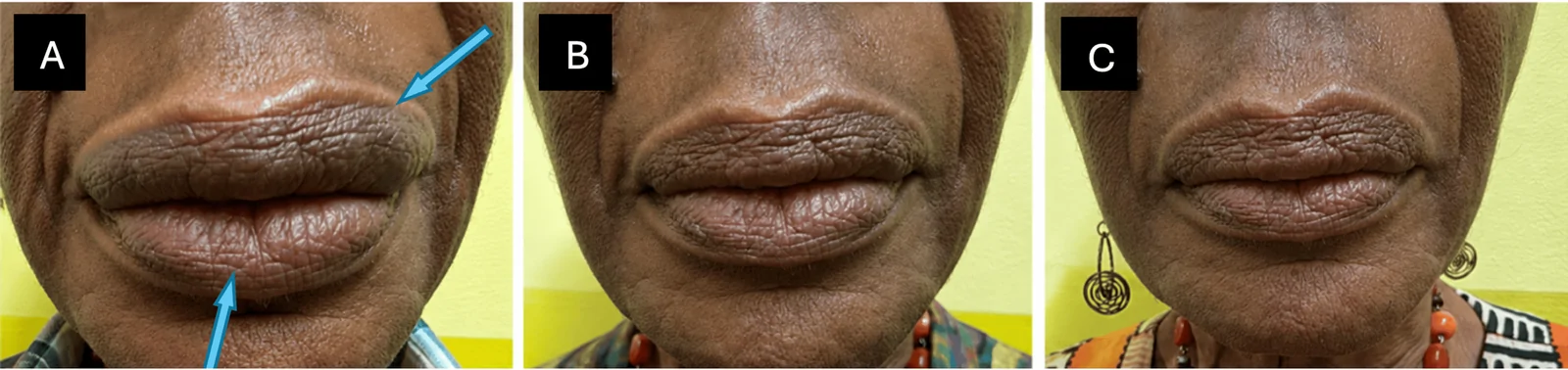
Serial clinical photographs demonstrating resolution of angioedema of the lip following discontinuation of spironolactone. (A) Day 1, at presentation to the primary health facility with marked isolated swelling of the lip. (B) Day 7 post discontinuation of spironolactone demonstrating substantial reduction in upper-lip edema. (C) Day 14 post discontinuation of spironolactone demonstrating complete resolution of the lip swelling.

On closer inspection of the face, the upper lip was visibly enlarged to approximately two to three times the size of the lower lip (Figure 1A). The affected area was non-tender, mildly warm and non-fluctuant, without erythema, skin breakdown or ulceration. There was no swelling of the tongue, floor of the mouth, soft palate, uvula or oropharynx, and there was no pooling of saliva. There was no periorbital edema, generalized facial swelling or asymmetry.

Initial laboratory investigations were unremarkable, with normal hematological parameters, renal function, serum electrolytes, and C-reactive protein (Table 1). Serum complement C4 concentration was 27.2 mg/dL (reference range, 10.0-40.0 mg/dL); however, C1-esterase inhibitor (C1-INH) antigenic level and functional activity assays were not available at the time of evaluation and therefore could not be performed.

**Table 1:** Laboratory parameters on initial presentation. BUN: blood urea nitrogen, AST: aspartate aminotransferase, ALT: alanine aminotransferase, CRP hs: high-sensitivity C-reactive protein, C4: complement component 4.

Blood parameters	Patient values	Reference range
Hemoglobin	13	11.7-15.5 g/dL
White blood cells	6.74	4.1-11.2 x 10^3 ^uL
Platelets	173	159-388 x 10^3 ^uL
Sodium	136	135.0-145.0 mmol/L
Potassium	3.7	3.5-5.1 mmol/L
Chloride	103.1	97.0-110.0 mmol/L
Creatinine	0.8	0.7-1.2 mg/dL
BUN	18	6-23 mg/dL
AST	35.3	5.0-40.0 IU/L
ALT	34	5.0-41.0 IU/L
CRP hs	0.12	0.100-0.500 mg/dL
C4	27.2	10.0-40.0 mg/dL

Given the isolated nature of the swelling, absence of urticaria or pruritus, lack of airway involvement, absence of an alternative medication or food trigger, and the temporal relationship between consistent spironolactone use and symptom onset, suspected spironolactone-associated angioedema was considered the most likely diagnosis. Other potential causes, including allergic reactions, infection, hereditary or acquired angioedema, and other medication-related reactions, were considered less likely based on the clinical history, examination, and available investigations.

Treatment

Spironolactone was discontinued immediately, and the patient was advised to avoid further exposure to the medication. Given the absence of tongue, oropharyngeal, or laryngeal involvement and the lack of respiratory or circulatory compromise, emergency airway intervention was not indicated. The patient was managed conservatively with close clinical observation and outpatient follow-up in seven days.

Outcome and follow-up

At follow-up seven days after discontinuation of spironolactone, the patient reported subjective improvement, with substantial reduction in the upper-lip swelling on clinical examination (Figure 1B). No new areas of facial, tongue, or oropharyngeal swelling had developed, and there were no respiratory or systemic symptoms. A further review was arranged one week later.

At Day 14 following discontinuation of spironolactone, the lip swelling had completely resolved without recurrence (Figure 1C). No subsequent episodes of angioedema were reported. The patient was counseled regarding the suspected association between spironolactone and angioedema and was advised not to restart spironolactone and a request for cardiology specialist review for optimization of medication.

Causality was assessed using the Naranjo Adverse Drug Reaction Probability Scale [9]. Application of the 10-item scale to the clinical findings in this case yielded a total score of 6, corresponding to a probable adverse drug reaction (Table 2). Rechallenge with spironolactone was not undertaken because of the potential risk of recurrent angioedema and airway compromise.

**Table 2:** Naranjo Adverse Drug Reaction Probability Scale, case-specific causality assessment of suspected spironolactone-associated angioedema.

No.	Naranjo criterion	Response	Score
1	Are there previous conclusive reports on this reaction?	No	0
2	Did the adverse event occur after the suspected drug was administered?	Yes	2
3	Did the adverse reaction improve when the drug was discontinued or a specific antagonist was administered?	Yes	1
4	Did the adverse reaction reappear when the drug was re-administered?	Not done	0
5	Are there alternative causes that could have caused the reaction?	No	2
6	Did the reaction reappear when a placebo was given?	Not done	0
7	Was the drug detected in blood or other fluids in concentrations known to be toxic?	Not done	0
8	Was the reaction more severe with increased dose or less severe with decreased dose?	Not known	0
9	Did the patient have a similar reaction to the same or similar drugs in any previous exposure?	No	0
10	Was the adverse event confirmed by any objective evidence?	Yes	1
	Total		6

## Discussion

This case highlights a probable association between spironolactone and isolated lip angioedema in a patient without urticaria, pruritus, airway involvement, or exposure to commonly implicated medications. Angioedema is well recognized with several drugs, particularly ACEi, with other agents affecting the renin-angiotensin system also implicated [5]. Although the angioedema in this patient was clinically limited in severity, with swelling confined to the upper lip and no tongue, oropharyngeal, laryngeal, respiratory, or hemodynamic involvement, angioedema is potentially a serious adverse drug reaction because progression to upper-airway involvement can result in airway obstruction and death [1,2]. Airway compromise is the principal life-threatening complication of angioedema, and clinical assessment should therefore focus particularly on the presence of tongue, floor of mouth, pharyngeal, or laryngeal edema, as well as symptoms such as dysphonia, hoarseness, stridor, or respiratory distress [1]. In the present case, the absence of these high-risk features supported conservative outpatient management; however, the potential for progression provides an important clinical rationale for prompt recognition, discontinuation of the suspected medication, and avoidance of rechallenge. Thus, while the immediate severity of the adverse drug reaction was limited, its potential seriousness warranted appropriate precautionary management.

A key feature was the temporal association between resumption of regular spironolactone therapy and symptom onset. The swelling persisted during continued exposure, followed by substantial improvement by Day 7 and complete resolution by Day 14 after discontinuation. This positive dechallenge provides important clinical support for a drug-related reaction and illustrates that the timing of actual medication exposure may be more informative than the original prescription date when assessing suspected adverse drug reactions.

Although isolated angioedema is not well established as an adverse effect of spironolactone, rare hypersensitivity reactions to the drug have previously been reported. Ghislain et al. described a spironolactone-associated drug reaction with eosinophilia and systemic symptoms (DRESS), with patch testing supporting drug hypersensitivity [6]. Bains et al. subsequently reported another case of spironolactone-associated DRESS, including recurrence following re-exposure [7]. Linear IgA bullous dermatosis associated with spironolactone has also been described [8]. Although these conditions are clinically distinct from angioedema, they demonstrate that spironolactone can rarely cause hypersensitivity reactions. The present case differs from these reports by its isolated lip involvement and absence of cutaneous or systemic features.

The mechanism underlying the present reaction remains uncertain. Drug-induced angioedema may occur through histamine- or bradykinin-mediated pathways [1,2]. Although bradykinin-mediated angioedema is well established with ACEi, the mechanism underlying spironolactone-associated angioedema has not been established. An immune-mediated reaction remains possible in view of previously reported spironolactone-associated hypersensitivity reactions [4,6,7], but the clinical findings do not permit definitive classification.

Alternative causes were considered clinically. The patient was not taking ACEi, ARBs, or NSAIDs and reported no new foods, infections, trauma, or procedures. There was no previous history of angioedema or family history suggestive of hereditary angioedema. Serum C4 was normal at 27.2 mg/dL. C1-INH antigenic level and functional activity were unavailable, preventing complete biochemical exclusion of hereditary or acquired C1-INH deficiency. Although the normal C4 concentration and clinical history made these diagnoses less likely, normal C4 alone does not completely exclude C1-INH deficiency.

Causality was assessed using the Naranjo Adverse Drug Reaction Probability Scale, which yielded a score of 6, consistent with a probable adverse drug reaction [9]. The score was supported by the temporal relationship, persistence during drug exposure, improvement following withdrawal, and absence of a more plausible alternative cause. Rechallenge was not performed because intentional re-exposure to a suspected cause of angioedema could result in a more severe reaction, including airway compromise. The Naranjo score therefore supports, but does not establish, causality and should be interpreted alongside the clinical course.

This report has several limitations. C1-INH antigenic and functional testing was unavailable, preventing complete biochemical exclusion of hereditary or acquired angioedema. Rechallenge was not undertaken for patient safety reasons, thereby limiting definitive causal confirmation. Additionally, the Naranjo scale is a general adverse drug reaction instrument and is not specific to angioedema. Accordingly, the reaction is classified as probable rather than certain.

## Conclusions

​​​​​​This case describes isolated lip angioedema without systemic or airway involvement occurring during regular spironolactone exposure. The temporal relationship, persistence of symptoms during continued exposure, subsequent resolution following discontinuation, and absence of another identified trigger support a probable association between spironolactone and the angioedema. However, definitive causation cannot be established because rechallenge was not performed and C1-INH antigenic and functional assays were unavailable, limiting the ability to completely exclude hereditary or acquired C1-INH deficiency. Clinicians should consider medication-related causes, including less commonly recognized agents, when evaluating unexplained isolated lip swelling and should establish the patient's actual pattern of medication exposure rather than relying solely on the prescribed medication history.

## References

[1] Trentman T, Misra L, Khurmi N (2016). Angioedema: classification, management and emerging therapies for the perioperative physician. Indian J Anaesth.

[2] Memon RJ, Tiwari V (2023). Angioedema. https://www.ncbi.nlm.nih.gov/books/NBK538489/.

[3] Patibandla S, Heaton J, Kyaw H (2023). Spironolactone. https://www.ncbi.nlm.nih.gov/books/NBK554421/.

[4] Win TS, Chaiyakunapruk N, Suwankesawong W, Dilokthornsakul P, Nathisuwan S (2015). Renin angiotensin system blockers-associated angioedema in the Thai population: analysis from Thai National Pharmacovigilance Database. Asian Pac J Allergy Immunol.

[5] Lin G, Chen R, Wen C, Li Z, Yan X, Li L (2025). Analyzing real-world adverse events of spironolactone with the FAERS database. PLoS One.

[6] Ghislain PD, Bodarwe AD, Vanderdonckt O, Tennstedt D, Marot L, Lachapelle JM (2004). Drug-induced eosinophilia and multisystemic failure with positive patch-test reaction to spironolactone: DRESS syndrome. Acta Derm Venereol.

[7] Bains A, Rajagopal SV, Rao M (2020). DRESS syndrome secondary to spironolactone with atypical presentation. Indian Dermatol Online J.

[8] Philip V, Ogunleye OO, Chukwu N, Rosenblum I, Collins S (2023). Linear IgA bullous dermatosis attributable to the use of spironolactone: a case report. Cureus.

[9] Naranjo CA, Busto U, Sellers EM (1981). A method for estimating the probability of adverse drug reactions. Clin Pharmacol Ther.

